# A Case Report of Hematometrocolpos in Two Adolescents: Divergent Clinical Presentations and Diagnostic Challenges

**DOI:** 10.7759/cureus.67675

**Published:** 2024-08-24

**Authors:** Nusrath M P, Lina Anwar

**Affiliations:** 1 Pediatric Emergency Medicine, Al Jalila Children's Speciality Hospital, Dubai, ARE; 2 Medical Education, Brunel Medical School, London, GBR

**Keywords:** urinary retention, adolescents, abdominal pain, imperforate hymen, hematometrocolpos

## Abstract

Hematometrocolpos is an infrequent congenital anomaly (Mullerian duct anomaly) that results in an imperforate hymen, followed by accumulation of menstrual blood in the vagina and or uterus in prepubertal girls results in retrograde menstruation. This commonly manifests as abdominal pain in premenarcheal pubescent girls. We discuss the case of two adolescent girls who presented to the emergency with lower abdominal pain, constipation, back pain, and/or urinary retention. They were found to have an imperforate hymen and hematometrocolpos. The diagnosis was made with the use of a genital examination and ultrasound. Hymenotomy was performed successfully in both cases and the patients recovered completely without complications. Failure to diagnose premenstrual girls presenting with lower abdominal pain and/or retention of urine with hematometrocolpos might lead to complications like infertility, endometriosis, tubal infections, adhesions, etc.

## Introduction

Hematometrocolpos is a disorder that occurs when menstrual blood causes a dilated vagina and uterus in the presence of a vaginal outflow obstruction, most commonly an imperforate hymen. The hymen is a remnant of mesodermal tissue that fails to perforate during embryonic development. Hematometrocolpos is an uncommonly diagnosed anomaly in the emergency department. In about 1 in every 2,000 females, the hymen does not perforate during development [1,2]. The incidence of imperforate hymen in term newborns ranges between 0.014% and 0.1% [3]. Patients with this abnormality are asymptomatic until menarche, with a common presentation of primary amenorrhea with cyclical pelvic pain, lower back pain, constipation, and urinary retention [4]. Hematometrocolpos may cause an abdominal/pelvic mass and bulging hymen. Hence, it should be suspected in premenarcheal females with sporadic pelvic and lower abdomen pain.

We present cases of hematometrocolpos in two adolescent girls caused by an imperforate hymen. Both manifested with lower abdominal pain and constipation. One of them experienced lower back pain while the other suffered acute urine retention. Ultrasound and physical examination can help prevent any complications and enable quick diagnosis and treatment.

## Case presentation

Case 1

A 12-year-old previously healthy female was escorted to the emergency department by her parents with a three-day history of bilateral intermittent pain in her lower abdomen. Her pain got worse after passing urine and radiated to the groin and upper thighs. She also complained of lower back pain, genital pain, and difficulty passing stool. This was not associated with bleeding or discharge per vagina, fever, vomiting, dysuria, or, diarrhea. She had not commenced her menstrual cycles. Further, she also denied engaging in sexual activity. She had been seen in another institution a few days prior with abdominal pain; the patient was diagnosed with constipation, advised on diet changes, and discharged on laxatives.

The patient was afebrile, and vitals were within normal limits. Her temperature was -37.1°C, with a heart rate of 111/mt, regular; respiration was 22/mt, regular, blood pressure was 122/86 mmHg, and oxygen saturation was 100% on room air. She had Tanner stage IV, a pain score of 5 out of 10. An examination of the abdomen revealed suprapubic fullness and tenderness. Other systemic examinations were unremarkable (Tables 1-3). No genitourinary examination had been performed, as the parents and patient refused.

**Table 1:** Full blood count for Case 1 MCV: mean corpuscular volume; MCH: mean corpuscular hemoglobin; MCHC: mean corpuscular hemoglobin concentration; RDW: red cell distribution width; MPV: mean platelet volume

COMPONENT	REFERENCE RANGE	RESULT
WBC count	5.0 - 13.0 10^3/uL	10.6
RBC count	4.00 - 5.20 10^6/uL	3.96
Hemoglobin	11.5 - 15.5 g/dL	11.2
Hematocrit	35.0 - 45.0 %	29.9
MCV	77.0 - 95.0 fL	75.5
MCH	25.0 - 29.0 pg	24.7
MCHC	31.5 - 34.5 g/dL	32.8
RDW	11.5 - 14.0 %	14.4
Platelet count	170 - 450 10^3/uL	389
MPV	7.4 - 10.4 fL	10.3
Neutrophil absolute	2.0 - 8.0 10^3/uL	6.23
Lymphocytes absolute	1.00 - 5.00 10^3/uL	3.19
Monocyte absolute	0.20 - 1.00 10^3/uL	0.98
Eosinophil absolute	0.10 - 1.00 10^3/uL	0.17
Basophil absolute	0.00 - 0.10 10^3/uL	0.04
Neutrophil %		58.7
Lymphocyte %		30.1
Monocyte %		9.2
Eosinophil %		1.6
Basophil %		0.4

**Table 2:** Basic metabolic panel for Case 1

COMPONENT	REFERENCE RANGE	RESULT
Sodium	136 - 145 mmol/L	137
Potassium	3.5 - 5.1 mmol/L	4.3
Chloride	97 - 107 mmol/L	105
Bicarbonate (HCO3)	17 - 27 mmol/L	23.0
Creatinine	0.52 - 0.69 mg/dL	0.51
Urea	15.622 - 40.66 mg/dL	19
Calcium	8.8 - 10.8 mg/dL	9.1
Glucose, random	73 - 112 mg/dL	104
C-reactive protein	0 - 5 mg/L	5

**Table 3:** Urinanalysis for Case 1 HPF: per high power field

COMPONENT	REFERENCE RANGE	RESULT
Urine color	Yellow	Yellow
Urine clarity	Clear	Clear
Urine pH	5.0 - 7.5	5.5
Urine protein	Negative	Trace
Urine glucose	Negative	Negative
Ketone	Negative	Negative
Bilirubin	Negative	Negative
Urobilinogen mg/dl	Negative	Negative
Nitrite	Negative	Negative
Leukocyte esterase	Negative	Negative
Blood by strip	Negative	Trace
Specific gravity	1.002 - 1.030	1.029
WBC/HPF	0 - 5	0-5
RBC/HPF	0 - 2	5-10
Epithelial cells/HPF	Not seen	Occasional

**Figure 1:**
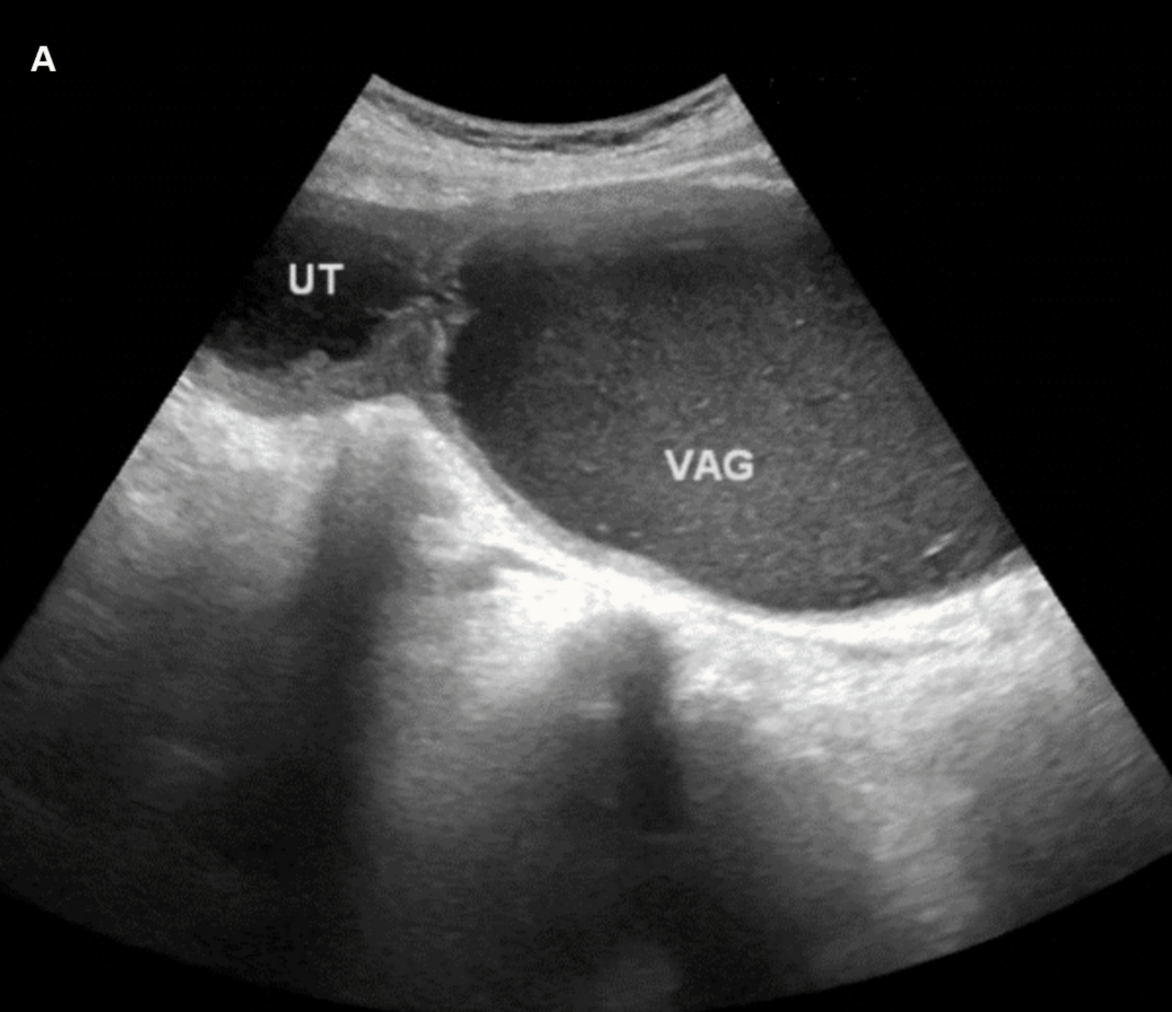
Ultrasound showing a markedly enlarged uterus and dilated vaginal canal with hypoechogenic material inside consistent with hematometrocolpos; uterine wall appears hyperechoic

**Figure 2:**
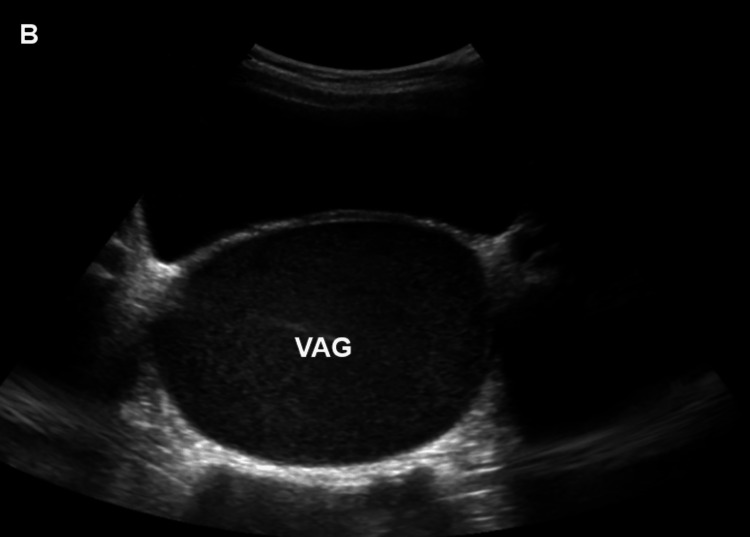
Ultrasound showing a markedly dilated vaginal canal with hypoechogenic collection

Blood count, inflammatory markers, serum electrolytes, renal function tests, and urinalysis were within normal limits. A dilated vaginal canal, a noticeably enlarged uterus (2x13.9x7.5 cm), thicker walls with homogenous internal echo patterns, and debris in the posterior wall, suggestive of hematometrocolpos, were all visible on an abdominal ultrasound scan. Both kidneys appear normal (Figures 1, 2).

Pediatric surgery was consulted. Subsequent examination of the external genitalia revealed a bulging imperforate hymen. The patient underwent a hymenotomy, which drained 1000 ml of dark blood and clots. The patient was discharged from the hospital the next day. Hymenal patency was confirmed at the one-month follow-up outpatient visit, and the child mentioned that she had experienced regular menstrual flow a few days prior.

Case 2

A 12-year-old previously healthy female was escorted to the emergency department with a three-day history of sporadic pain in the lower abdomen and urinary retention for 12 hours. Her pain was bilateral and not associated with fever, vomiting, dysuria, vaginal bleeding, or discharge. She experienced constipation as well. She had not commenced her menstrual cycles. Further, she denied engaging in sexual activity.

The patient was afebrile and vitals were within normal limits. Her temperature was 37.3°C, heart rate was 100/mt, regular; respiration was 20/mt, regular; blood pressure was 120/83 mmHg, and oxygen saturation was 100% in room air. She had a Tanner stage IV, pain score of 8 out of 10. An examination of the abdomen revealed a palpable suprapubic mass that reached the umbilicus and was tender, which may have been caused by a distended bladder. Other systemic examinations were unremarkable. The pelvic exam revealed a blue, bulging imperforate hymen. The urinary bladder was catheterized and drained 1000 ml of urine. When the abdomen was reevaluated, there was mild suprapubic tenderness and fullness in the lower abdomen.

**Figure 3:**
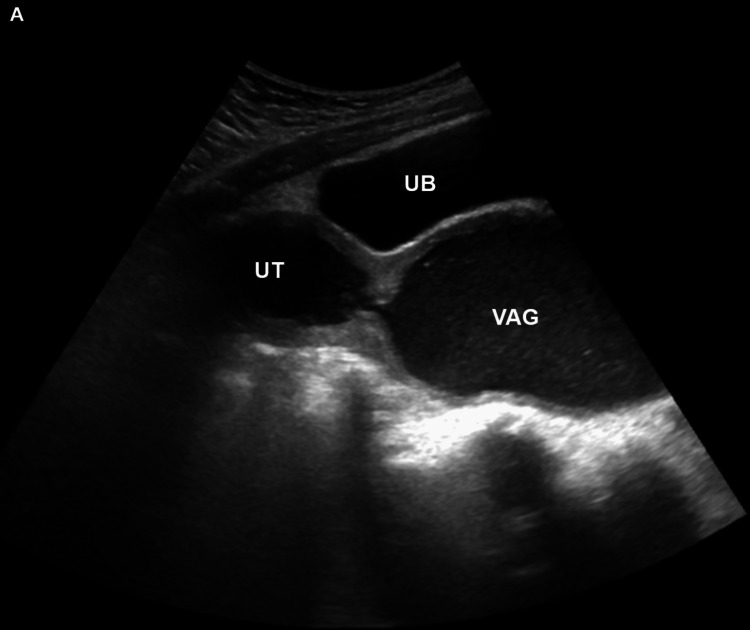
Ultrasound showing an enlarged uterus and distended vaginal canal with homogeneous internal echoes and debris suggestive of hematometrocolpos

**Figure 4:**
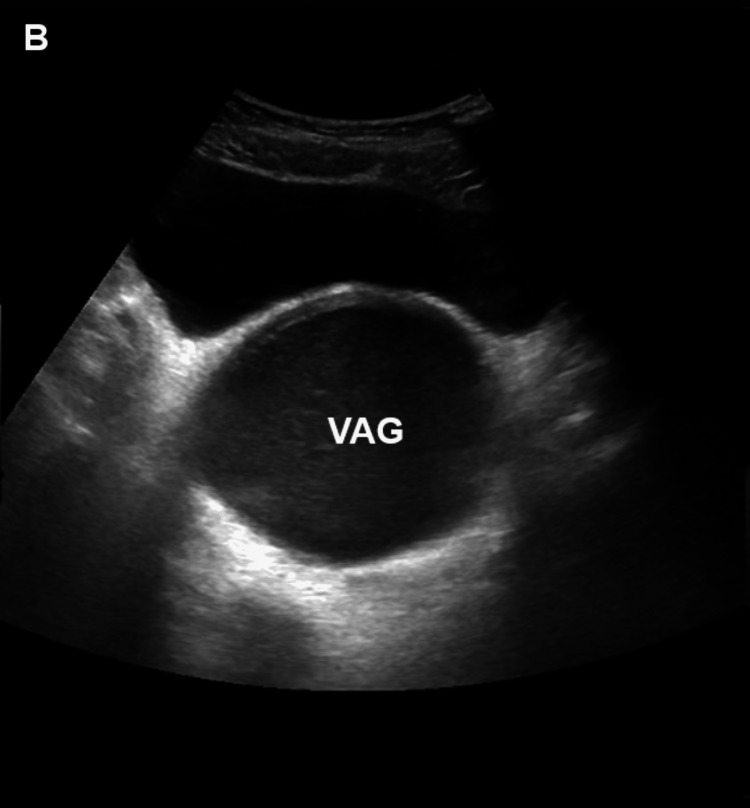
Ultrasound showing a distended vaginal canal with hypoechoic material

Blood count, inflammatory markers, serum electrolytes, renal function tests, and urinalysis were within normal limits (Tables 4-6). Abdominal ultrasonography showed features of hematometrocolpos, enlarged uterus (4.2x1.8x.5 cm) with distended vaginal canal (16x7.7x10cm), thicker walls with homogeneous internal echos, and debris with mild left kidney hydronephrosis (Figures 3, 4).

**Table 4:** Full blood count for Case 2 MCV: mean corpuscular volume; MCH: mean corpuscular hemoglobin; MCHC: mean corpuscular hemoglobin concentration; RDW: red cell distribution width; MPV: mean platelet volume

COMPONENT	REFERENCE RANGE	RESULT
WBC count	5.0 - 13.0 10^3/uL	7.8
RBC count	4.00 - 5.20 10^6/uL	5.03
Hemoglobin	11.5 - 15.5 g/dL	12.3
Hematocrit	35.0 - 45.0 %	38.3
MCV	77.0 - 95.0 fL	76.1
MCH	25.0 - 29.0 pg	24.5
MCHC	31.5 - 34.5 g/dL	32.1
RDW	11.5 - 14.0 %	13.2
Platelet count	170 - 450 10^3/uL	280
MPV	7.4 - 10.4 fL	11.9
Neutrophil absolute	2.0 - 8.0 10^3/uL	5.22
Lymphocyte absolute	1.00 - 5.00 10^3/uL	2.03
Monocyte absolute	0.20 - 1.00 10^3/uL	0.45
Eosinophil absolute	0.10 - 1.00 10^3/uL	0.04
Basophil absolute	0.00 - 0.10 10^3/uL	0.04
Neutrophil %		67.1
Lymphocyte %		26.1
Monocyte %		5.8
Eosinophil %		0.5
Basophil %		0.5

**Table 5:** Basic metabolic panel for Case 2

COMPONENT	REFERENCE RANGE	RESULT
Sodium	136 - 145 mmol/L	132
Potassium	3.5 - 5.1 mmol/L	3.5
Chloride	97 - 107 mmol/L	105
Bicarbonate (HCO3)	17 - 27 mmol/L	18.0
Creatinine	0.52 - 0.69 mg/dL	0.42
Urea	15.622 - 40.66 mg/dL	11
Calcium	8.8 - 10.8 mg/dL	9.2
Glucose, random	73 - 112 mg/dL	121
C-reactive protein	0 - 5 mg/L	1

**Table 6:** Urinanalysis for Case 2 HPF: per high power field

COMPONENT	REFERENCE RANGE	RESULT
Urine color	Yellow	Yellow
Urine clarity	Clear	Clear
Urine pH	5.0 - 7.5	5.6
Urine protein	Negative	Negative
Urine glucose	Negative	Negative
Ketone	Negative	Negative
Bilirubin	Negative	Negative
Urobilinogen mg/dl	Normal	Normal
Nitrite	Negative	Negative
Leukocyte esterase	Negative	Negative
Blood by strip	Negative	Negative
Specific gravity	1.002 - 1.030	1.021
WBC/HPF	0 - 5	0-5
RBC/HPF	0 - 2	0-2

Pediatric surgery was consulted. Following a hymenotomy, 700 ml of black blood and clots were removed from the patient. Two months later, her menstruation returned to normal, and she recovered without any complications. Her hydronephrosis was resolved.

## Discussion

Congenital obstructions of the vaginal or uterine outflow may occur at different levels and can manifest with varying clinical presentations [5]. Menstrual blood accumulates in the vaginal cavity in hematocolpos. It happens due to the vaginal outflow tube being obstructed. Imperforate hymen, transverse vaginal septum, vaginal atresia, hemi-vaginal atresia (Herlyn Werner Wunderlich syndrome), cervicovaginal atresia, aberrant vaginal opening, cloacal malformation, and acquired vaginal stenosis are all frequently linked to vaginal outflow blockage. Despite being uncommon abnormalities of the female genital canal, hematocolpos and hydrometrocolpos have been documented to occur at an incidence between 3.8% and 0.1% [6]. 

The hymen is an embryologic remnant of mesodermal tissue. During adolescence, a buildup of successive menstruation occurs in the uterus and vagina due to failure to perforate during embryonic development. While an imperforate hymen normally occurs sporadically, reports of familial cases have been reported. It is believed that either autosomal dominant or autosomal recessive transmission occurs [7]. Although an imperforate hymen is often a solitary occurrence, McKusick-Kaufman syndrome and Bardet-Biedl syndrome can also be associated with it [8]. Congenital anorectal malformations, multicystic dysplastic kidneys, and polydactyly are other potentially related anomalies.

An imperforate hymen and hematometrocolpos are rare but significant diagnoses to consider when adolescent female patients present with complaints of abdominal pain without menstruation. Until menarche, most patients with this congenital anomaly are asymptomatic. The majority of patients experience symptoms during menstruation when the endometrial tissue and retained blood accumulate and cause the distention of the uterus (hematometra), vagina (hematocolpos), or both (hematometrocolpos). Due to the condition's varying presentation, diagnosis might be challenging and take longer. Patients frequently present with pelvic pain, abdominal pain, and vomiting. Back pain, constipation, urinary retention, and incontinence are less frequent symptoms [2,9]. Hemotometocolpos physical exam findings include a palpable mass in the pelvis and a protruding hymen in the genital examination.

In our instance, one of the adolescents presents with acute urinary retention in addition to constipation and abdominal pain. When a young female arrives in the emergency with acute urine retention, hematometrocolpos is usually an uncommon cause [4,9]. Between 3% and 46% of individuals with an imperforate hymen present with acute urine retention [10,11]. The retained hematoma in the vagina may compress the urethra or irritate the sacral plexus, which are the possible causes of imperforate hymen producing acute urine retention. Urinary outflow blockage may also arise from the mechanical action of the vaginal hematoma changing the angle formed between the bladder neck and urethra.

Similar cases had been reported previously in the literature, and there is a greater risk of misdiagnosing a palpable pelvic mass [12]. In our case, the first occurrence was identified as constipation and treated as such. This emphasizes the significance of performing an ultrasound scan and genital inspection on adolescent females who have not attained menarche but exhibit symptoms of abdominal pain and urine retention.

The preferred imaging modality to evaluate for hematometrocolpos is ultrasonography. Usually, an ultrasound shows a large, smooth-walled, hypoechoic mass posterior to the bladder. The uterus may appear normal or dilated, depending on how much the pelvic organs have been dilated. The uterine blood and endometrial tissue have a hypoechoic appearance [13,14]. In the case of hematometrocolpos, measuring the fluid volume may help the surgical team prepare for the procedure. Additionally, hydronephrosis and free fluid in the abdomen from uterine perforation are consequences of hematometrocolpos that can be evaluated with ultrasound.

If hematocolpos is confirmed by ultrasonography, additional imaging is not necessary. If the diagnosis is unclear, to rule out urogenital abnormalities like a transverse or longitudinal vaginal septum, an obstructed uterine horn, distant vaginal atresia, or cervical atresia, magnetic resonance imaging is advised [12].

Treatment of an imperforate hymen consists of surgical repair. The tissue and blood are removed by making a cut the membrane next to the hymenal ring. Serious consequences include pelvic endometriosis, renal failure due to hydronephrosis, tubal infection and adhesion, and infertility if the diagnosis is missed or treatment is delayed [13,15]. In our case, the hymenotomy went well, and the patient recovered completely and without any complications.

## Conclusions

An imperforate hymen, although an uncommon diagnosis, should be considered in premenarcheal adolescent girls who experience lower abdominal pain, back pain, or urine retention. If a genital examination is not done on teenage females who haven’t attained menarche and come to the emergency room complaining of lower abdomen pain or trouble urinating, the diagnosis of imperforate hymen may go unnoticed. Therefore, emergency physicians need to be more aware of the possibility of imperforate hymen when they examine teenage females who have intermittent lower abdomen pain, constipation, or urine retention. It is also important to emphasize that ultrasound examinations are crucial for assessing young females. Early intervention with hymenotomy prevents any future complications.
